# The prognostic value of age for invasive lobular breast cancer depending on estrogen receptor and progesterone receptor-defined subtypes: A NCDB analysis

**DOI:** 10.18632/oncotarget.5844

**Published:** 2015-10-28

**Authors:** Jieqiong Liu, Kai Chen, Kai Mao, Fengxi Su, Qiang Liu, Lisa K. Jacobs

**Affiliations:** ^1^ Guangdong Provincial Key Laboratory of Malignant Tumor Epigenetics and Gene Regulation, Department of Breast Surgery, Breast Tumor Center, Sun Yat-sen Memorial Hospital, Sun Yat-sen University, Guangzhou, China; ^2^ Department of Surgery, Johns Hopkins University School of Medicine, Baltimore, MD, USA; ^3^ Guangdong Provincial Key Laboratory of Malignant Tumor Epigenetics and Gene Regulation, Department of General Surgery, Sun Yat-sen Memorial Hospital, Sun Yat-sen University, Guangzhou, China; ^4^ Department of Medicine, Johns Hopkins University School of Medicine, Baltimore, MD, USA

**Keywords:** age, prognostic value, ER/PR-defined subtypes, lobular breast cancer, national cancer database

## Abstract

**Purpose:** We aimed to assess the effect of age on survival according to estrogen receptor (ER) and progesterone receptor (PR)-defined lobular breast cancer subtype in a wide age range.

**Methods:** 43,230 invasive lobular breast cancer women without comorbidities diagnosed between 2004 and 2011 in the National Cancer Database (NCDB) were analyzed. The effects of age on overall survival (OS) among different age groups were evaluated by log-rank test and Cox proportional model.

**Results:** Multivariate analysis showed that patients diagnosed at both young (<35 years) and old (&ge;70 years) ages had worse prognosis compared with those in the middle ages. We further analyzed the interaction between age and molecular subtype for predicting OS: in ER+PR+ subtype, the HR of OS declined with age from 1.55 (95% CI, 1.08&ndash;2.22; *P* = 0.019) in the group younger than 35 years to 1.38 (1.02&ndash;1.86; *P* = 0.036) in the 35&ndash;39 group, but increased with age to 10.1 (8.49&ndash;11.94; *P* < 0.001) in the group older than 79. While in ER+PR- and ER-PR- subtypes, the HRs showed no statistical differences among women diagnosed before 60 (*P* > 0.1); and in ER-PR+ subgroup, the HRs were similar in patients younger than 70 (*P* > 0.1); thus, the plots of HRs in these three subtypes remained steady until the age of 60 or 70.

**Conclusions:** Our findings identified that the effect of age on OS in lobular breast cancer varied with ER/PR-defined subtypes. Personalized treatment strategies should be developed to improve outcomes of breast cancer patients with different ages and ER/PR statuses.

## INTRODUCTION

Breast cancer is known to be a heterogeneous disease, which exhibits distinct clinical presentations, aggressiveness, treatment response, as well as outcomes among different subtypes of breast cancer. Accurate prognostic factors play a critical role in treatment decision-making during the management of this life-threating disease. Besides the classic biologic predictors, age at diagnosis has been proved as an independent prognostic factor in several studies [1–9]. Although most of the studies showed that the survival difference according to age was observed only in patients with hormone receptor (HR)–positive tumors [5, 7–11], these studies just investigated the association between age and outcomes in a narrow age range (e.g. only compared patients <35 years with those ≥35 years), and they included very few lobular carcinoma cases.

Invasive lobular carcinoma accounts for approximately 10% of all breast cancer cases, and it differs from invasive ductal carcinoma [12]. Several retrospective studies showed that invasive lobular breast cancer was less responsive than ductal carcinoma to chemotherapy [13–15]; and a recent analysis of BIG 1–98 trial demonstrated that the benefit of adjuvant letrozole was greater for postmenopausal women diagnosed with lobular carcinoma versus ductal carcinoma [16]. We hypothesized that age may have a more complicated prognostic impact on survival outcomes than prior realized in this particular histological type of breast cancer.

Indeed, little is known about the prognostic value of age according to ER/PR-defined subtypes for invasive lobular breast cancer [17]. A single-institutional retrospective study with small sample size reported age ≥70 years was an independent predictor of reduced overall survival (OS) for invasive lobular breast cancer patients [17]. But no studies have addressed whether impact of age on survival would differ by ER/PR-defined subtypes in lobular breast cancer so far. It is well documented that ER and PR are significant predictive molecular factors in breast cancer, and ER/PR positive cancers have much favorable outcomes than ER/PR negative diseases. Recent studies demonstrated that ER+PR+, ER+PR- and ER-PR+ breast cancers had distinct clinicopathological characteristics and survival outcomes although they all belong to HR positive disease [18–20]. Lack of either ER or PR expression was associated with significantly worse survival compared with ER+PR+ breast cancer [20]. Therefore, the prognostic significance of age for breast cancer might vary with different ER/PR subtypes (ER+/PR+, ER+/PR-, ER-/PR+, and ER-PR-).

Here, we analyzed a large national cohort of invasive lobular breast cancer patients using the National Cancer Database (NCDB) to assess the prognostic value of age on OS of different ER/PR-defined breast cancer subtypes in a wide age range. The large sample size and available systemic therapy information in this database enabled us to better quantify the impact of age on survival and provide high-quality evidence for individualized treatment decision-making of lobular breast cancer.

## RESULTS

### Patient characteristics

Of the 43,230 primary invasive lobular breast cancer patients with no comorbid conditions included in this study, 27,962 (64.7%) patients had ER+PR+ tumors, 5,000 (11.5%) patients had ER+PR-disease, 585 (1.4%) women had ER-PR+ cancers, and 9,683 (22.4%) women had ER-PR- subtype of tumors. Table 1 shows the demographic and tumor characteristics for each ER and PR-defined molecular subtype. The differences for all the demographic and clinicopathological variables among the four subtypes were statistically significant (*P* < 0.001 for all comparisons), which might be partially explained by the large sample size of the study. ER-PR+ and ER-PR- subtypes had more patients who were diagnosed at a very young age (<35 years) compared with ER+PR+ or ER+PR- subgroup (*P* < 0.001, Table 1). Obviously, ER+PR+ and ER+PR- patients had more low grade (well-differentiated) and small tumor (T1) than ER-PR+ or ER-PR- women (*P* < 0.001, Table 1). Regarding treatment characteristics, ER-PR+ and ER-PR- women underwent significantly more chemotherapy than ER+PR+ or ER+PR- patients; and ER-PR- patients received extremely less endocrine therapy compared with the other three subtypes (*P* < 0.001, Table 1).

**Table 1:** Demographic and clinicopatholgical characteristics of the study cohort

Characteristics	ER/PR-defined Subtypes								*P*
	ER+PR+		ER+PR-		ER-PR+		ER-PR-		
	No.	%	No.	%	No.	%	No.	%	
Age									<0.001
<35 yrs	526	1.9	86	1.7	33	5.6	368	3.8	
35–39 yrs	982	3.5	174	3.5	30	5.1	518	5.3	
40–49 yrs	6,059	21.7	734	14.7	150	25.6	2,213	22.9	
50–59 yrs	7,209	25.8	1,452	29.0	167	28.6	2,829	29.2	
60–69 yrs	6,577	23.5	1,274	25.5	113	19.3	2,098	21.7	
70–79 yrs	4,455	15.9	839	16.8	65	11.1	1,129	11.7	
>79 yrs	2,154	7.7	441	8.8	27	4.6	528	5.4	
Race									<0.001
White	24,647	88.9	4,242	84.8	454	77.6	7,718	79.7	
Black	2,119	7.6	572	11.5	106	18.1	1,560	16.1	
Other	1,196	4.3	186	3.7	25	4.3	405	4.2	
Year of diagnosis									<0.001
2004–2007	14,939	53.4	2,881	57.6	368	62.9	5,436	56.1	
2008–2011	13,023	46.6	2,119	42.4	217	37.1	4,247	43.9	
Histologic grade									<0.001
Well	7,316	25.5	803	16.1	30	5.1	205	2.1	
Moderately	13,617	48.7	2,079	41.6	126	21.5	1,649	17.0	
Poorly	7,209	25.8	2,118	42.3	429	73.3	7,829	80.9	
Tumor stage									<0.001
T1	16,141	57.7	2,647	52.9	283	48.4	3,703	38.2	
T2–T4	11,821	42.3	2,353	47.1	302	51.6	5,980	61.8	
Node stage									<0.001
N0	19,216	68.7	3,434	68.7	399	68.2	6,718	69.4	
N1	6,691	23.9	1,119	22.4	132	22.6	2,072	21.4	
N2	1,558	5.6	329	6.6	22	20.2	622	6.4	
N3	497	1.8	118	2.3	11	10.1	271	2.8	
Surgery type									<0.001
Lumpectomy	18,135	64.9	3,069	61.4	363	62.1	5,659	58.4	
Mastectomy	9,872	35.1	1,931	38.6	222	37.9	4,024	41.6	
Radiotherapy									<0.001
No	9,978	35.7	1,918	38.4	217	37.1	3,866	39.9	
Yes	17,984	64.3	3,082	61.6	368	62.9	5,817	60.1	
Chemotherapy									<0.001
No	16,067	57.5	2,388	47.8	159	27.2	2,161	22.3	
Yes, single-agent	342	1.2	91	1.8	15	2.6	141	1.5	
Yes, multi-agent	10,789	38.6	2,358	47.2	380	65.0	6,960	71.9	
Yes, unknown type	764	2.7	163	3.3	31	5.3	421	4.3	
Hormone therapy									<0.001
No	5,906	21.1	1,308	26.2	287	49.1	9,306	96.1	
Yes	22,056	78.9	3,692	73.8	298	50.9	377	3.9	

Abbreviations: ER, estrogen receptor; PR, progesterone receptor

### Survival analysis among different age groups according to ER/PR-defined subtype

**Figure 1:**
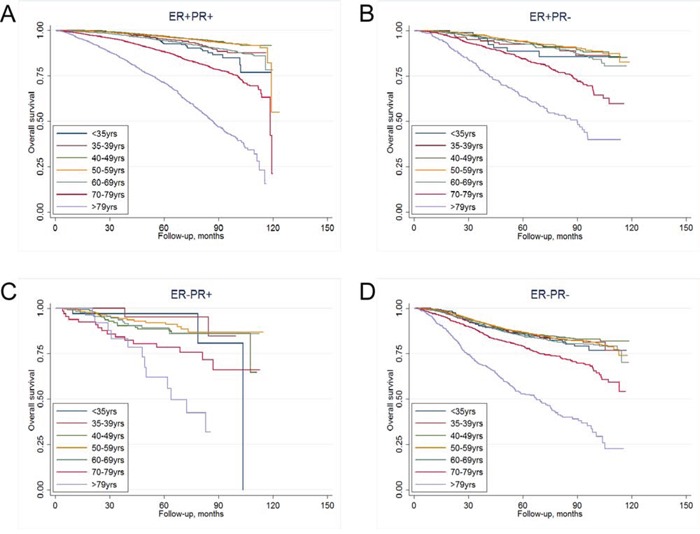
Kaplan-Meier curves of overall survival in different age groups for A. ER+PR+ subtype, B. ER+PR- subtype, C. ER-PR+ subtype, and D. ER-PR- subtype

The last follow-up date in the study was December 31th, 2013. With a median follow-up of 58.2 months for the entire study cohort (*n* = 43,230), a total of 4,511 (10.4%) patients died. Kaplan–Meier curves were used to investigate OS in different age groups among the four ER/PR-defined subtypes (Figure 1A–1D). The patients who were diagnosed after 79 years old had the worst overall survival rate in all of the four subtypes, whereas the age group that had the best prognosis varied with different ER/PR-defined subtypes (*P* < 0.001, Figure 1 and Table 2). Among ER+PR+ and ER+PR- patients, women who were 40–59 years of age at diagnosis had the best survival rate; while in ER-PR+ and ER-PR- subgroups, patients who were diagnosed before 70 years showed similar survival rates (Figure 1 and Table 2).

**Table 2:** Cumulative 5-year OS rates of different combinations of age and ER/PR subtypes

	ER/PR-defined Subtypes			
	ER+PR+	ER+PR-	ER-PR+	ER-PR-
	5-year OS rate (%)	5-year OS rate (%)	5-year OS rate (%)	5-year OS rate (%)
Age, years				
<35	92.7	88.6	96.9	85.7
35–39	95.1	92.5	95.1	86.4
40–49	96.8	93.9	88.6	86.4
50–59	96.6	93.9	92.0	86.8
60–69	94.3	92.7	89.1	84.5
70–79	88.4	84.3	78.3	79.1
>79	71.3	63.2	61.8	52.7

Abbreviations: OS, overall survival; ER, estrogen receptor; PR, progesterone receptor

**Figure 2:**
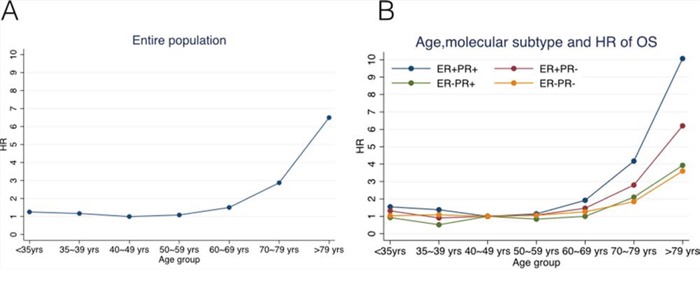
Hazard ratios (HRs) of overall survival changing with age for A. the entire study population, B. ER+PR+, ER+PR-, ER-PR+, and ER-PR- subgroups of patients using Cox proportional hazard model

Multivariate Cox proportional hazard model was used to further study the effect of age on OS. We found that ER/PR-defined subtype, age at diagnosis, race, histologic grade, pathologic tumor and nodal stage, receiving of radiotherapy, chemotherapy and hormone therapy were significantly associated with OS (*P* < 0.04 for all comparisons, Table 3). Lack of either ER or PR expression was associated with significantly worse survival compared with ER+PR+ subtype (*P* < 0.01 for all comparisons). And surgery type (lumpectomy *vs*. mastectomy ± reconstruction) had a marginal effect on OS (*P* = 0.051, Table 3). We used the 40–49-year-old group as the reference for the different age groups in the multivariate analysis based on results of the univariate analysis. In the entire cohort of patients, the hazard ratio (HR) of OS declined with age, from 1.25 (95% CI, 1.01–1.56; *P* = 0.040) in the group younger than 35 years old to 1.17(95% CI, 0.97–1.41; *P* = 0.099) in the 35–39 group, and kept statistically flat through 1.09 (95% CI, 0.97–1.21; *P* = 0.135) in the 50–59 group, but then elevated to 6.50 (95% CI, 5.80–7.28; *P* < 0.001) in the group older than 79 years (Figure 2A).

**Table 3:** Multivariate analysis of OS for the whole study population

Factors	HR	95% CI	*P*
Age, years			
<35	1.26	1.01–1.56	**0.040**
35–39	1.17	0.97–1.41	0.099
40–49	Reference		
50–59	1.09	0.97–1.21	0.135
60–69	1.51	1.35–1.68	**<0.001**
70–79	2.88	2.58–3.20	**<0.001**
>79	6.50	5.80–7.28	**<0.001**
Race			
White	Reference		
Black	1.32	1.21–1.45	**<0.001**
Other	0.56	0.48–0.72	**<0.001**
ER/PR-defined subtype			
ER+PR+	Reference		
ER+PR-	1.23	1.12–1.97	**<0.001**
ER-PR+	1.48	1.18–1.86	**0.001**
ER-PR-	1.37	1.25–1.50	**<0.001**
Histologic grade			
Well differentiated	Reference		
Moderately differentiated	1.28	1.16–1.42	**<0.001**
Poorly differentiated	1.66	1.49–1.85	**<0.001**
Tumor stage			
T1	Reference		
T2–T4	1.74	1.63–1.86	**<0.001**
Nodal stage			
N0	Reference		
N1	1.55	1.44–1.67	**<0.001**
N2	2.75	2.47–3.05	**<0.001**
N3	4.30	3.75–4.93	**<0.001**
Surgery type			
Lumpectomy	Reference		
Mastectomy	0.93	0.86–1.00	**0.051**
Radiotherapy			
No	Reference		
Yes	0.68	0.63–0.73	**<0.001**
Chemotherapy			
No	Reference		
Yes, single-agent	0.76	0.64–0.91	**0.002**
Yes, multi-agent	0.67	0.52–0.85	**0.001**
Yes, unknown type	0.67	0.62–0.73	**<0.001**
Hormone therapy			
No	Reference		
Yes	0.62	0.57–0.67	**<0.001**

Abbreviations: OS, overall survival; HR, hazard ratio; CI, confidence interval; ER, estrogen receptor; PR, progesterone receptor

### Survival analysis according to age and ER/PR-defined subtype using different interaction terms

To investigate the potential interaction between ER/PR-defined subtype and age as predictors of OS, we performed pairwise comparisons (by multivariate Cox proportional model) between distinct combinations of age and ER/PR subtypes regarding OS (Table 4). The trend of HR changing with age at diagnosis differed by ER/PR-defined subtype (Figure 2B). In the ER+PR+ group, the HR of OS declined with age from 1.55 (95% CI, 1.08–2.22; *P* = 0.019) in the group younger than 35 years old to 1.38 (95% CI, 1.02–1.86; *P* = 0.036) in the 35–39 group, but increased with age to 10.1 (95% CI, 8.49–11.94; *P* < 0.001) in the group older than 79. The curve of the HRs in this subtype declined slightly first and then elevated sharply (Figure 2B). While in ER+PR- and ER-PR- subtypes, the HRs showed no statistical differences among patients who were diagnosed before 60 years (*P* > 0.1 for all), which was consistent with the previous log-rank test; and in ER-PR+ subgroup, the HRs of patients younger than 70 years old were not significantly different (*P* > 0.1 for all), which was also in accordance with the log-rank analysis. Thus, the plots of the HRs in these three subtypes remained steady until the patients reached an older age (60 or 70 years, Figure 2B).

**Table 4:** Pairwise comparisons between distinct combinations of age and ER/PR subtypes regarding OS^#^

	ER/PR-defined Subtypes							
	ER+PR+		ER+PR-		ER-PR+		ER-PR-	
	HR (95% CI)	*P*	HR (95% CI)	*P*	HR (95% CI)	*P*	HR (95% CI)	*P*
Age, years								
<35	1.55 (1.08–2.22)	**0.019**	1.31 (0.62–2.77)	0.473	0.93 (0.27–3.21)	0.908	1.04 (0.77–1.40)	0.819
35–39	1.38 (1.02–1.86)	**0.036**	0.91 (0.50–1.64)	0.747	0.52 (0.12–2.29)	0.385	1.09 (0.84–1.42)	0.522
40–49	Reference		Reference		Reference		Reference	
50–59	1.14 (0.95–1.36)	0.148	1.07 (0.76–1.49)	0.702	0.84 (0.42–1.68)	0.619	1.06 (0.90–1.24)	0.494
60–69	1.92 (1.62–2.27)	**<0.001**	1.47 (1.05–2.05)	**0.024**	1.00 (0.48–2.09)	0.996	1.26 (1.07–1.49)	**0.006**
70–79	4.17 (3.54–4.92)	**<0.001**	2.80 (2.02–3.88)	**<0.001**	2.10 (1.01–4.35)	**0.046**	1.84 (1.54–2.20)	**<0.001**
>79	10.07 (8.49–11.94)	**<0.001**	6.20 (4.40–8.74)	**<0.001**	3.93 (1.62–9.56)	**0.003**	3.60 (2.96–4.38)	**<0.001**

Abbreviations: ER, estrogen receptor; PR, progesterone receptor; OS, overall survival; HR, hazard ratio; CI, confidence interval

# All results were adjusted by race, histologic grade, tumor/node stage, surgery type, radiotherapy, chemotherapy and hormone therapy using Cox proportional harzard model.

## DISCUSSION

In this large, registry-based, national cohort study using data from NCDB, we quantified the prognostic impact of age on OS of different ER/PR-defined lobular breast cancer subtypes in a wide age range. After adjusting for known breast cancer prognostic factors and multiple treatment variables, we demonstrated that patients who were diagnosed with invasive lobular breast cancer at both very young (<35 years) and old (≥70 years) ages had significantly worse prognosis compared with those in the middle age groups. The more important finding was that different patterns of the effect that age had on OS, according to the ER/PR-defined breast cancer subtype were observed in our study.

To the best of our knowledge, the present study is the first and largest study that assessed the prognostic values of age on survival according to the ER/PR-defined molecular subtype in lobular breast cancer. Regarding the impact of age on outcomes in breast cancer, most of the studies found that the survival difference based on age was observed only in patients with HR-positive cancers [5–8, 10, 11], but these studies just investigated the association between age and survival in a narrow age range, and they included limited cases of lobular carcinomas. A recent research using the SEER database observed different patterns of the effect of age on BCSS in HR+ and HR- breast cancers, but the study was restricted to ductal carcinomas, and their results had inherent weakness due to lack of information on chemotherapy and hormone therapy [23]. A small retrospective study conducted at the European Institute of Oncology reported that age ≥70 years was an independent prognostic factor of reduced OS for lobular breast cancer patients, however, there was very few (*n* = 9) young cases (<35 years) in their study cohort, and no in-depth analysis has been performed to assess the effect of age on OS according to the ER/PR status [17]. Although our findings that women with both very young and old ages had worse OS was not totally consistent with this European study, we think our results were much more generalizable because of our significantly larger sample size and multi-institutional design. Our observations might be explained by several reasons: i) breast cancer onset at an early age might be correlated with elevated familial risk and genetic predisposition (e.g. BRCA1/2 mutations) that would result in suboptimal outcomes; ii) older patients (perticularly those diagnosed after 79 years) may have significantly shorter life expectancy, and they are more likely to develop multiple complications during chemotherapy or endocrine therapy than younger patients; iii) older patients might be treated with significantly less standard adjuvant radiotherapy, chemotherapy or hormone therapy, for instance, they may receive reduced treatment cycles and dosage, shorter duration of long-term endocrine therapy [24, 25].

More interestingly, we identified that the effect of age at diagnosis on OS differed by ER/PR-defined subtype. The HRs for the ER+PR+ subtype declined slightly first and then elevated sharply when plotted against age, whereas the HRs for the ER-PR- patients remained steady until they reached an elder age (>60 years old). This is partially in accordance with the findings of above-mentioned SEER study, although their study population was invasive ductal breast cancer patients [23]. Indeed, our new finding was that we observed distinct impact of age on survival between ER+PR+ subtype and single hormone receptor positive subtypes (ER-PR+ or ER+PR-). Unlike ER+PR+ subtype, the HRs for the ER-PR+ and ER+PR- subgroups were constant until they reached an older age of 70 and 60, respectively. This might be due to the totally different biological features and clinical prognosis among these three ER/PR-defined subtypes. A recent report in Nature found that in the presence of agonist ligands, PR associates with ER to direct ERα chromatin binding events within breast cancer cells, resulting in a unique gene expression programme that is associated with good clinical outcomes [26]. And several clinical studies have investigated that ER+PR+, ER+PR- and ER-PR+ subtypes showed distinct clinicopathological characteristics and outcomes although they all belong to luminal breast cancer [18–20]. Furthermore, we noticed that the HRs of the older patients (≥70 years) elevated more sharply and greatly in ER+PR+ subtype compared with the other subgroups. It suggests that older age as a prognostic factor for unfavorable outcomes, seems to play a more important role in ER+PR+ lobular breast cancer. In a word, our study demonstrated that the impact of age on OS of lobular breast cancer in ER+PR- and ER-PR+ subtypes were more likely to be similar with that in ER-PR- subtype rather than ER+PR+ subgroup, and young age was not a predictor for worse overall survival in ER+PR-, ER-PR+ or ER-PR- lobular breast cancer. Further studies are required to define the underlying mechanisms.

In addition, besides the well-defined independent prognostic factors of lobular breast cancer including age, race, histologic grade, pathologic tumor and nodal stage, receiving of radiotherapy, chemotherapy and hormone therapy, for the first time, we found that the surgery type had a marginal effect on OS (*P* = 0.051). Thus, lobular carcinoma patients who received breast-conserving surgery seemed to have better prognosis than those who were treated with mastectomy. Several recent population-based studies that included lobular breast cancer cases also identified an OS benefit of breast-conserving surgery *vs*. mastectomy, however, the percentages of lobular carcinomas in these studies were either very small (≤10%) or unknown, and no subset analysis was performed to assess the impact of surgery type on survival in lobular cancers [27–29]. Further randomized controlled studies addressing this issue are warranted to confirm our findings.

Despite several strengths of this study including its multicenter large sample size, refined subgroup analyses, novel insights on the prognostic value of age according to ER/PR-defined subtype in a very wide age range, some limitations should be acknowledged. First, our study has inherent weakness because of its nonrandomized, retrospective nature, there might be some selection biases of patient characteristics among comparative groups. Second, the NCDB suffers from lack of Ki67, human epidermal growth factor receptor 2 (HER2), menopausal status and lymphovascular invasion information, as well as the information regarding pleomorphic lobular carcinomas, which are known to be associated with survival. However, in a retrospective analysis of a large randomized controlled trial of women with early-stage HER2-positive breast cancer, age was not significantly correlated with risk of early recurrence or prediction of benefit from trastuzumab therapy [30]. Third, due to the lack of central testing of hormone receptor status, patients in the ER/PR-defined subtypes might be misgrouped, which may cause potential biases to our results. Finally, although we excluded patients with comorbid conditions in our analysis, other confounders may complicate the association between age and OS, particularly among older patients. And we cannot assess the effect of age on BCSS due to the limitation of NCDB.

## MATERIALS AND METHODS

### Patient population

We used data from the NCDB, which is a national hospital-based cancer registry jointly sponsored by the American College of Surgeons and the American Cancer Society, and collects data on about 70% of newly diagnosed breast cancer cases from approximately 1,450 Commission on Cancer-approved hospitals across the United States. Data are coded and reported according to nationally established protocols coordinated under the auspices of the North American Association of Central Cancer Registries.

A total of 146,290 pure lobular breast cancer cases (International Classification of Diseases for Oncology, 3rd edition [ICD-O-3] histology codes 8520 [21]) diagnosed between 2004 and 2011, were identified. This study was limited to women 18 years or older who had a pathologic diagnosis of T1–4, N0–3, M0 (AJCC) pure lobular breast cancer. Patients with neoadjuvant therapy, unknown pathologic tumor, node or AJCC stage, unknown or undifferentiated/anaplastic tumor grade, unknown ER/PR status, distant metastatic disease, bilateral breast cancers, or prior malignancy were excluded. We also excluded patients with comorbid conditions because of the significant impact of comorbidities on OS. This resulted in a cohort of 43,230 patients for the final analysis.

Data within the NCDB were rendered anonymous, so the study was exempt from review by the Johns Hopkins Medicine Institutional Review Board, and no consent was needed in this study.

### Statistical analysis

The study outcome was OS, which was calculated from the date of diagnosis to the date of death from any cause, with surviving patients censored at date of last contact. To investigate the impact of age on OS, we treated age at diagnosis as a categorical variable classified into the following age groups: younger than 35 years, 35–39 years, 40–49 years, 50–59 years, 60–69 years, 70–79 years, and older than 79 years. The demographic statistics of patient included age at diagnosis, race, and year of diagnosis. The clinicopathological characteristics included histologic grade, laterality, pathologic tumor/node stage, number of examined regional lymph nodes, ER and PR status. The treatment information comprised surgery type, receiving of radiotherapy, and receiving of systemic chemotherapy or hormone therapy. The lobular breast cancer subtypes were defined as ER+PR+, ER+PR-, ER-PR+, and ER-PR- according to the ER and PR statuses of tumor. ER or PR positive groups included those with borderline results [22].

To assess whether there was an interaction between age and ER/PR-defined subtypes in predicting OS, we created an interaction term. Pairwise comparisons were performed between different combinations of age and ER/PR-defined subtypes. Patient demographics, clinicopathological and treatment characteristics by breast cancer subtypes were compared using the χ2 test. The Kaplan–Meier method was used to analyze survival curves. Multivariate Cox proportional hazard model was applied to determine whether the independent prognostic effect of age on OS vary with distinct ER/PR-defined subtypes of lobular breast cancer.

Statistical analyses were conducted using STATA 13.0 software (StataCrop LP, College Station, TX). All statistical tests were two-sided, and the statistical significance was defined as *P* < 0.05.

## CONCLUSIONS

In summary, we demonstrated that the prognostic effect of age on OS in lobular breast cancer varied with different ER/PR-defined subtypes. The HRs for ER+PR+ subtype declined slightly first and then elevated sharply when plotted against age, whereas the HRs for ER+PR-, ER-PR+, and ER-PR- subgroups remained steady until they reached an older age (>60 or 70 years). Personalized management strategies should be developed to improve outcomes of breast cancer patients with different ages and ER/PR statuses. For example, young (<40yrs) ER+PR+ invasive lobular breast cancer women might be undertreated if they receive only chemotherapy or endocrine therapy. Adjuvant chemotherapy followed by endocrine therapy may be recommended to these patients. Moreover, our findings can help clinicians with the patient consultation about the long term prognosis according to their age and ER/PR status. Further prospective studies are needed to confirm our findings.
